# Microcystin-LR exposure induces cartilage degeneration and dysregulates osteoarthritis-associated targets: an integrative computational and experimental study

**DOI:** 10.3389/fphar.2026.1910273

**Published:** 2026-10-26

**Authors:** Zhifang Tang, Zuyong Feng, Zhiyu Liang, Shijie Liao, Xiaofei Ding

**Affiliations:** 1 Department of Orthopedic Trauma and Hand Surgery, The First Affiliated Hospital of Guangxi Medical University, Nanning, China; 2 Collaborative Innovation Centre of Regenerative Medicine and Medical Bioresource Development and Application Co-Constructed by the Province and Ministry, Guangxi Medical University, Nanning, China

**Keywords:** environmental toxicology, machine learning, microcystin-LR, molecular dynamics simulation, network toxicology, osteoarthritis

## Abstract

**Introduction:**

Microcystin‐LR (MC‐LR), a prevalent cyanotoxin in eutrophic freshwater, is becoming a more widespread environmental contaminant as global warming promotes cyanobacterial blooms, and its health effects may extend beyond its well-documented hepatotoxicity.

**Methods:**

Here, we integrated network toxicology, machine learning, molecular dynamics simulation (MDS), and in vitro and in vivo experiments to investigate the role of MC‐LR in osteoarthritis (OA). A total of 368 predicted MC‐LR targets were intersected with OA‐related differentially expressed genes from five batch-corrected Gene Expression Omnibus (GEO) datasets.

**Results:**

Weighted gene co-expression network analysis (WGCNA) identified a disease-correlated red module (r = 0.61, p = 2.3e–13), and least absolute shrinkage and selection operator (LASSO) regression narrowed the candidates to a six‐gene diagnostic signature (FAP, IGF1R, IGF1, MMP9, CRABP2, and ITGA4), with a combined area under the curve (AUC) of 0.910. CIBERSORT analysis indicated altered macrophage polarization and T‐cell redistribution within the osteoarthritic microenvironment. Molecular docking and 100-ns MDS showed that MC‐LR formed thermodynamically favorable and stable complexes with the five upregulated core targets, with FAP exhibiting the strongest binding affinity (–10.3 kcal/mol). In human chondrocytes, MC-LR exposure was cytotoxic (IC_50_ = 13.09 μM) and upregulated the mRNA levels of CRABP2, FAP, IGF1, ITGA4, and MMP9, consistent with the in silico predictions. Intra-articular injection of MC‐LR in mice induced dose-dependent knee cartilage degeneration, with matrix loss and elevated OARSI scores.

**Discussion:**

Together, these findings associate MC‐LR exposure with cartilage degeneration under experimental conditions and implicate PI3K‐Akt signaling, focal adhesion, and extracellular matrix remodeling as candidate mechanisms, extending the recognized effects of cyanotoxins beyond the liver and highlighting chronic pollutant exposure as an underrecognized potential risk factor for degenerative joint disease.

## 1 Introduction

Osteoarthritis (OA) is the most prevalent chronic degenerative joint disease affecting more than 300 million people and is a leading cause of disability in aging populations (Steinmetz et al., 2023). OA is characterized by progressive cartilage degradation, subchondral bone remodeling, osteophyte formation, and low-grade synovial inflammation (Yang et al., 2024). Recent paradigm shifts in systems medicine emphasize that systemic biomarker landscapes and multi-omics profiling play an indispensable role in capturing the multi-tissue pathological continuum, early diagnostic stratification, and molecular crosstalk across joint diseases (Li et al., 2026). Within this framework, integrating high-dimensional molecular signatures provides crucial insights into OA-associated disease hubs. Beyond traditional risk factors, environmental toxins may contribute to the pathogenesis of OA (Berenbaum et al., 2018; Leung et al., 2017). Environmental pollutants are thought to injure the joint through several converging routes, including oxidative stress, chondrocyte senescence and apoptosis, an inflammatory response, and impaired extracellular-matrix production, ultimately reducing chondrocyte viability (Berenbaum et al., 2018; Skalny et al., 2024).

Among cyanotoxins, microcystin-LR (MC-LR) is the most abundant and toxic microcystin congener produced by *Microcystis*, *Planktothrix*, and *Anabaena* (Huisman et al., 2018; Paerl and Huisman, 2008). MC-LR has been detected in drinking water, recreational water, aquatic food chains, and aerosols near bloom-affected lakes (Carmichael and Boyer, 2016; Merel et al., 2013). With global warming and accelerating eutrophication driving the worldwide expansion of cyanobacterial blooms, the contamination of drinking water and aquatic products by MC-LR has emerged as a pressing environmental and public health concern (Huisman et al., 2018; Paerl and Huisman, 2009). Human exposure occurs via ingestion, dermal contact, and inhalation (Chorus and Welker, 2021). Reported concentrations span the WHO provisional lifetime drinking-water guideline value of 1 μg/L (approximately 0.001 μM) for microcystin-LR (World Health Organization, 2020), up to tens of μg/L in bloom-affected surface water (e.g., total cellular microcystins of 20–44 μg/L in Lake Taihu, China) (Sakai et al., 2013). Microcystins are also detectable in human serum at the nanogram-per-millilitre level in chronically exposed populations (mean ≈ 0.39 ng/mL in Lake Chaohu fishermen) (Chen et al., 2009; Zheng et al., 2017), underscoring the relevance of chronic low-level exposure.

MC-LR toxicity is mediated by the potent, irreversible inhibition of protein phosphatases (PPs) PP1 and PP2A, central regulators of cell proliferation, apoptosis, cytoskeletal organization, and inflammation (MacKintosh et al., 1990; Shi, 2009). To date, however, MC-LR research has focused almost exclusively on hepatotoxicity, whereas its potential extrahepatic effects, particularly on the musculoskeletal system, are largely unexplored. Although hepatic injury dominates the current toxicological literature, MC-LR affects the kidneys and the intestine, as well as the reproductive and nervous systems (Chen et al., 2021; Ge et al., 2024; Zhao et al., 2020). Importantly, emerging evidence indicates that PP2A is not merely a housekeeping phosphatase in cartilage. It also regulates chondrocyte differentiation, extracellular matrix (ECM) homeostasis, and the anabolic–catabolic balance, and suppresses catabolic effectors such as matrix metalloproteinase (MMP)-13 that drive cartilage degradation (Wei et al., 2025; Zákány et al., 2002). Given that MC-LR is a potent inhibitor of both PP1 and PP2A, chronic exposure could plausibly tip this balance toward cartilage breakdown; however, this hypothesis has never been systematically tested. No prior study has applied an integrative network toxicology framework to dissect MC-LR–OA mechanisms, leaving a critical knowledge gap at the intersection of environmental toxicology and degenerative joint disease.

We therefore combined network toxicology, molecular docking and dynamics, and interpretable machine learning (LASSO with SHAP) with *in vitro* and *in vivo* experiments. Given that MC-LR is a potent inhibitor of PP1/PP2A, phosphatases that help maintain cartilage homeostasis, we hypothesized that MC-LR may act on OA-associated molecular targets and be intrinsically toxic to articular cartilage. To examine this, we predicted MC-LR targets and their overlap with OA-associated genes, prioritized and characterized candidate targets by docking, molecular dynamics, and an interpretable machine-learning model, and then evaluated the effects of MC-LR on chondrocyte viability and candidate-gene expression *in vitro* and on knee cartilage integrity in a mouse model. The overall study design is shown in Figure 1.

**FIGURE 1:**
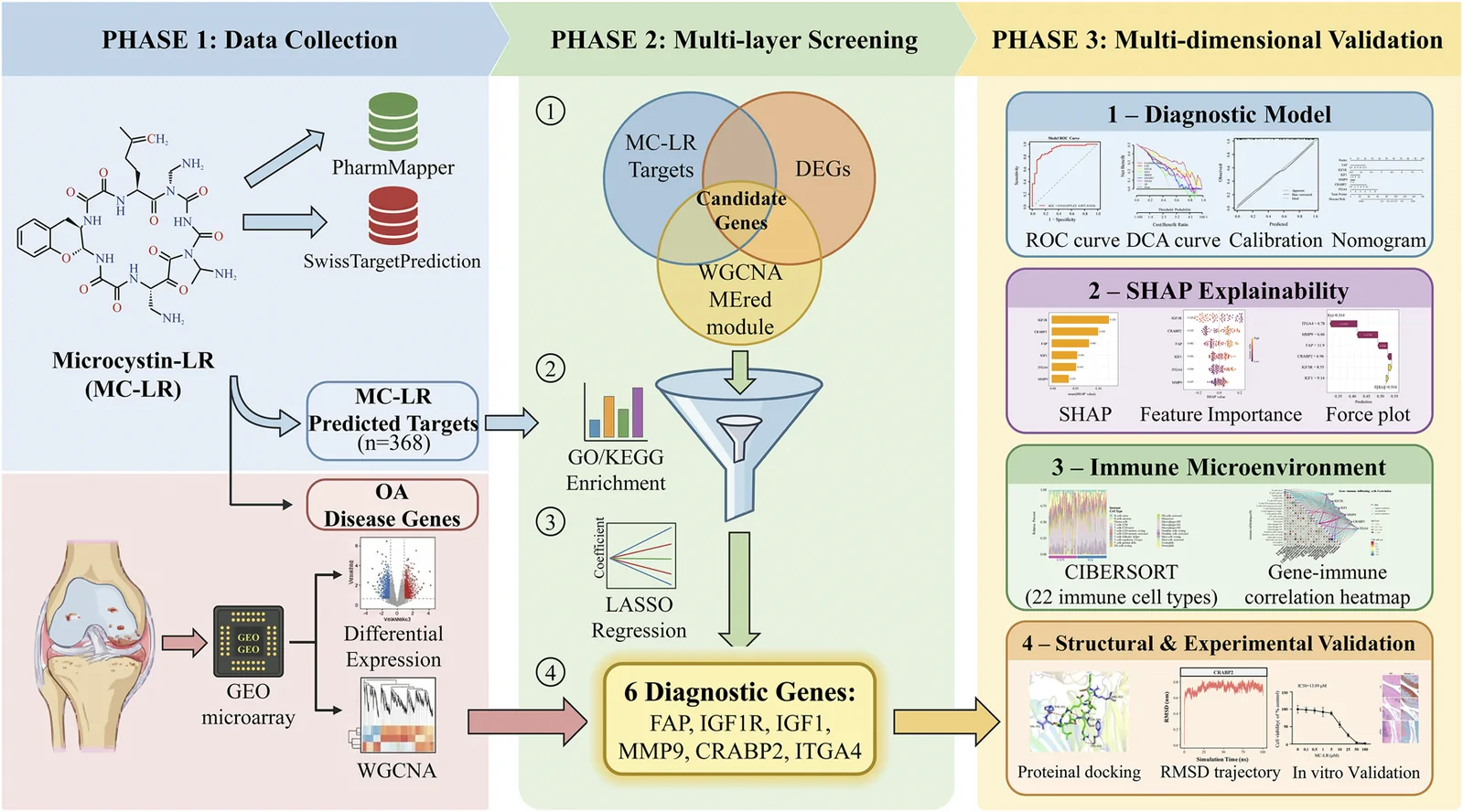
Overall study design and integrative analytical workflow.

## 2 Materials and methods

### 2.1 Data collection and target prediction

The canonical SMILES of MC-LR (C_49_H_74_N_10_O_12_) was retrieved from PubChem. Targets were predicted using PharmMapper and SwissTargetPrediction (*Homo sapiens*, probability ≥0.1). OA-related genes were obtained from the GEO datasets GSE114007, GSE117999, GSE12021, GSE55235, and GSE55457 (Table 1). Differential expression was performed using *limma* (adjusted p < 0.05, |log_2_fold change| > 1.0), and batch effects were removed using ComBat (*sva*). Principal component analysis (PCA) was used to visualize sample distributions before/after correction, and intersections were identified using *VennDiagram*.

**TABLE 1:** Characteristics of the five osteoarthritis gene-expression datasets.

Dataset	Tissue source	Platform	Sample size (OA/control)	Disease stage	Control source
GSE114007	Knee articular cartilage	RNA-seq (Illumina HiSeq 2000, GPL11154; NextSeq 500, GPL18573)	20/18	End-stage OA (knee arthroplasty; OA score 4 vs. control score 1)	Normal knee cartilage from tissue banks (no joint disease or trauma)
GSE117999	Knee articular cartilage (non–weight-bearing intercondylar notch)	Microarray (Agilent SurePrint G3 human GE v3 8 × 60 K, GPL20844)	10/10	End-stage OA (TKA)	Non-OA cartilage from arthroscopic partial meniscectomy (meniscus-tear) knees
GSE12021	Synovial membrane	Microarray (Affymetrix HG-U133A, GPL96; HG-U133B, GPL97)	10/9	Not reported	Normal synovial membrane
GSE55235	Synovial membrane	Microarray (Affymetrix HG-U133A, GPL96)	10/10	Not reported	Healthy synovial membrane
GSE55457	Synovial membrane	Microarray (Affymetrix HG-U133A, GPL96)	10/10	Not reported	Healthy synovial membrane

### 2.2 WGCNA and functional enrichment

Disease-associated co-expression modules were identified using WGCNA (R package (Langfelder and Horvath, 2008)), and module–trait correlations were identified using Pearson with p < 0.05. Genes in the most disease-correlated module were intersected with MC-LR targets and DEGs to obtain core candidates. Gene Ontology (GO) and Kyoto Encyclopedia of Genes and Genomes (KEGG) enrichment were performed using clusterProfiler (Yu et al., 2012). Enrichment significance was determined using the hypergeometric test with Benjamini–Hochberg correction (adjusted p-value <0.05).

### 2.3 Machine learning and diagnostic evaluation

LASSO logistic regression (Friedman et al., 2010) was used for feature selection. Data were split 70/30 (stratified), and the optimal λ was determined using 10-fold cross-validation (glmnet). The six selected genes were combined into a nomogram and evaluated using AUC, calibration curves, and decision curve analysis (DCA). Model interpretability was assessed using SHAP (Lundberg and Lee, 2017), including feature importance, beeswarm, density, dependence, and waterfall/force plots. Analyses were conducted using scikit-learn 1.2.0 and shap 0.41.0 in Python 3.9.

### 2.4 Immune microenvironment analysis

Immune-cell infiltration patterns were analyzed using the CIBERSORT algorithm to deconvolute 22 immune cell types from bulk RNA-seq data (Newman et al., 2015). Gene expression profiles were normalized and filtered to only include genes with expression values >0 in at least 50% of samples. CIBERSORT was run with 1,000 permutations, and p-value <0.05 was considered significant for deconvolution results. Correlation analysis between immune cell proportions and diagnostic genes was performed using Pearson correlation coefficient. The differential infiltration of immune cell types between the control (CON; n = 57) and OA (n = 60) groups was compared using the Wilcoxon rank-sum test. A correlation network between the six core biomarkers and immune-cell abundances was constructed as an exploratory, hypothesis-generating layer to complement the differential-expression and enrichment results, and nominal p-values are reported accordingly. Because the CIBERSORT LM22 reference signature was derived from peripheral blood, its deconvolution of solid joint tissue, and of the mixed cartilage and synovial samples used here, provides approximate relative estimates that should be interpreted with caution.

### 2.5 Molecular docking and MDS

Five significantly upregulated targets in the OA group (*FAP*, *IGF1*, *MMP9*, *CRABP2*, and *ITGA4*) were selected for molecular docking and MDS. The three-dimensional structures of the following target proteins were retrieved from Protein Data Bank (PDB): FAP (PDB: 1Z68), IGF1 (PDB: 1GZR), MMP9 (PDB: 4H3X), CRABP2 (PDB: 1CBS), and ITGA4 (PDB: 4WJK). Protein structures were prepared by removing water molecules and the initial ligands with PyMOL, whereas proteins were prepped using AutoDock Tools 1.5.7 for hydrogen addition, charge estimation, and merging nonpolar hydrogens. Molecular docking was performed using AutoDock Vina (Trott and Olson, 2009). The results were visualized using Discovery Studio and PyMOL. The lowest binding energies (kcal/mol) were used to evaluate binding affinities.

MDS was performed using GROMACS 2022.3 with the AMBER99SB-ILDN force field for the protein and the TIP3P model for explicit water molecules (Abraham et al., 2015). Ligand parameterization was performed following a quantum chemical approach: the initial 3D structure of the ligand (MC-LR) was geometry-optimized using Gaussian at the B3LYP/6-31G* level, followed by single-point electrostatic potential (ESP) calculations at the HF/6-31G* level. Restrained electrostatic potential (RESP) atomic partial charges were subsequently derived using Multiwfn (Lu and Chen, 2012). Force field parameters and topology files for the ligand under the General AMBER Force Field (GAFF) were generated via Sobtop (Lu, 2022). Each protein–ligand complex was immersed in a cubic box with a minimum distance of 1.0 nm between the complex and the box edge under periodic boundary conditions (PBC). The system was neutralized and brought to a physiological ionic strength of 150 mM NaCl. Energy minimization was executed using the steepest descent algorithm until maximum force reached $<1000{\;\text{kJ}\;\text{mol}}^{-1}{\;\text{nm}}^{-1}$ . The system was then equilibrated sequentially under an NVT ensemble for 100 ps at 300 K using the velocity-rescale (V-rescale) thermostat (τ_t = 0.1 ps), followed by an NPT ensemble for 100 ps at 1.0 bar using the Parrinello–Rahman barostat (τ_p = 2.0 ps). Production MD simulations were carried out for 100 ns with a integration time step of 2 fs. All bonds involving hydrogen atoms were constrained using the LINCS algorithm, and long-range electrostatic interactions were computed via the Particle Mesh Ewald (PME) method with a cutoff distance of 1.0 nm. Trajectory frames were saved every 10 ps. Post-simulation analyses were performed using built-in GROMACS utilities to evaluate root mean square deviation (RMSD), root mean square fluctuation (RMSF), radius of gyration ( $R_{g}$ ), and intermolecular hydrogen bond counts. Complex stability was confirmed when RMSD trajectories plateaued and fluctuated within a stable range (typically below ∼1 nm) during the final production window. To explore conformational energy basins, the Gibbs free-energy landscape (FEL) was constructed using the gmx sham module based on the joint probability distribution $P\left(q_{1},q_{2}\right)$ along the first two principal components (PC1 and PC2) derived from the covariance matrix of backbone $C_{\alpha }$ atoms, calculated as:

$$G\left(q_{1},q_{2}\right)=-k_{B}T\ln P\left(q_{1},q_{2}\right)$$

where $k_{B}$ is the Boltzmann constant and T is the simulation temperature (300 K).

### 2.6 Cell culture

Human chondrocytes were purchased from Procell Life Science & Technology Co., Ltd. (Wuhan, China). Cells were cultured in Dulbecco’s Modified Eagle Medium (Gibco, Thermo Fisher Scientific, Waltham, MA, United States) supplemented with 10% fetal bovine serum (Gibco, Thermo Fisher Scientific, United States) and 1% penicillin–streptomycin (Servicebio, Wuhan, China) and maintained at 37 °C in a humidified atmosphere containing 5% CO_2_. The culture medium was replaced every 2 days, and cells in the logarithmic growth phase were used for subsequent experiments.

### 2.7 Cell viability assay

The effect of MC-LR (MCE) on the viability of human chondrocytes was determined using the Cell Counting Kit-8 (CCK-8; Cat. No. HY-K0301, MedChemExpress [MCE], United States) assay. Cells were seeded into 96-well plates at a density of 1 × 10^4^ cells per well and allowed to adhere overnight at 37 °C in a humidified 5% CO_2_ incubator (Heracell 150i, Thermo Fisher Scientific, Waltham, MA, United States). The cells were treated with MC-LR at concentrations ranging from 0 to 100 µM for 24 h. At the end of the treatment period, 10 µL of CCK-8 reagent was added to each well, and the plates were incubated at 37 °C for an additional 2 h. Absorbance at 450 nm was measured using a TriStar^2^ LB 942 multimode microplate reader (Berthold Technologies GmbH & Co. KG, Bad Wildbad, Germany). Cell viability is expressed as a percentage relative to the vehicle control, and the half-maximal inhibitory concentration (IC_50_) was determined using four-parameter nonlinear regression (Hill equation) in GraphPad Prism 9.0 (Le Berre et al., 2022). This concentration range and the 24 h exposure were selected to establish an acute concentration-response relationship and to determine the cytotoxic potency (IC_50_) of MC-LR, and were not intended to reproduce environmentally encountered exposure levels.

### 2.8 RNA extraction and reverse-transcription quantitative polymerase chain reaction (RT-qPCR)

RT-qPCR was performed to quantify the mRNA expression of target genes. Human chondrocytes were seeded into 6-well plates at a density of approximately 2 × 10^5^ cells per well and allowed to adhere overnight. Cells were then treated with MC-LR at the IC_50_ concentration (13.09 µM) for 24 h. After treatment, total RNA was extracted using TRIzol reagent (Thermo Fisher Scientific, Waltham, MA, United States) according to the manufacturer’s instructions, with sequential chloroform extraction, isopropanol precipitation, and 75% ethanol washing. RNA concentration and purity were assessed using spectrophotometry (A_260_/A_280_ ratio). Complementary DNA was synthesized from 1 µg of total RNA using a RevertAid reverse-transcription kit (Thermo Fisher Scientific; Cat. No. K1691). Quantitative PCR was performed using SYBR Green Master Mix (Sangon Biotech; Cat. No. B690016) on an Applied Biosystems 7,500 Real-Time PCR System (Thermo Fisher Scientific, Waltham, MA, United States) with gene-specific primers (Table 2). Relative mRNA expression was normalized to *GAPDH* and calculated using the 2^−ΔΔCT^ method (Livak and Schmittgen, 2001). Primer sequences are listed in Table 2. All experiments were performed in triplicate, and statistical comparisons between groups were made using Student’s t-test (p < 0.05 was considered significant).

**TABLE 2:** Primer sequences for RT-qPCR.

Gene	Forward primer (5′→3′)	Reverse primer (5′→3′)
*FAP*	ATG​AGC​TTC​CTC​GTC​CAA​TTC​A	AGA​CCA​CCA​GAG​AGC​ATA​TTT​TG
*IGF1*	GCT​CTT​CAG​TTC​GTG​TGT​GGA	GCC​TCC​TTA​GAT​CAC​AGC​TCC
*CRABP2*	ATC​GGA​AAA​CTT​CGA​GGA​ATT​GC	AGG​CTC​TTA​CAG​GGC​CTC​C
*ITGA4*	CAC​AAC​ACG​CTG​TTC​GGC​TA	CGA​TCC​TGC​ATC​TGT​AAA​TCG​C
*MMP9*	TGT​ACC​GCT​ATG​GTT​ACA​CTC​G	GGC​AGG​GAC​AGT​TGC​TTC​T
*GAPDH*	GGA​GCG​AGA​TCC​CTC​CAA​AAT	GGC​TGT​TGT​CAT​ACT​TCT​CAT​GG

### 2.9 Animal experiment and histological evaluation

Male C57BL/6J mice (8 weeks old; Experimental Animal Center of Guangxi Medical University) were maintained under standard conditions. The study was approved by the Ethics Committee of the First Affiliated Hospital of Guangxi Medical University (No. 2026-E0503). Mice were randomized into three groups, (n = 3 mice/6 knees per group): saline control, low-dose MC-LR (5 μM), and high-dose MC-LR (50 μM). These intra-articular doses were chosen to deliver a defined local bolus for evaluating acute cartilage responses and, like the *in vitro* concentrations, are not intended to model chronic environmental exposure.

Under 1% sodium pentobarbital anesthesia (50 mg/kg, i.p.), bilateral intra-articular injections of 5 μL saline or MC-LR (99.34% purity; MedChemExpress) were performed via the patellar ligament into the intercondylar space. After the experimental period, knee joints were harvested, fixed, decalcified, and paraffin-embedded. Sections were stained with Hematoxylin and Eosin (HE) and Safranin O–Fast Green to assess cartilage integrity and matrix loss. Cartilage damage was quantified using the OARSI 0–6 grading system. Statistical significance was determined using the Kruskal–Wallis test followed by Dunn’s multiple-comparison test (p < 0.05).

## 3 Results

### 3.1 MC-LR target identification and functional enrichment

The three-dimensional structure of MC-LR is shown in Figure 2A. In total, 368 targets were predicted: 264 (71.7%) were unique to PharmMapper, 79 (21.5%) were unique to SwissTargetPrediction, and 25 (6.8%) were shared (Figure 2B). GO enrichment analysis (Figure 2C) highlighted wound healing and response to peptide (biological process [BP]); vesicle lumen and cell–substrate junction (cellular component [CC]); and protein serine/threonine/tyrosine kinase activity and carboxylic acid binding (molecular function [MF]). KEGG (Figure 2D) analysis identified PI3K-Akt signaling, proteoglycans in cancer, focal adhesion, and Ras signaling as the top pathways, providing a systems-level link between MC-LR and cartilage homeostasis.

**FIGURE 2:**
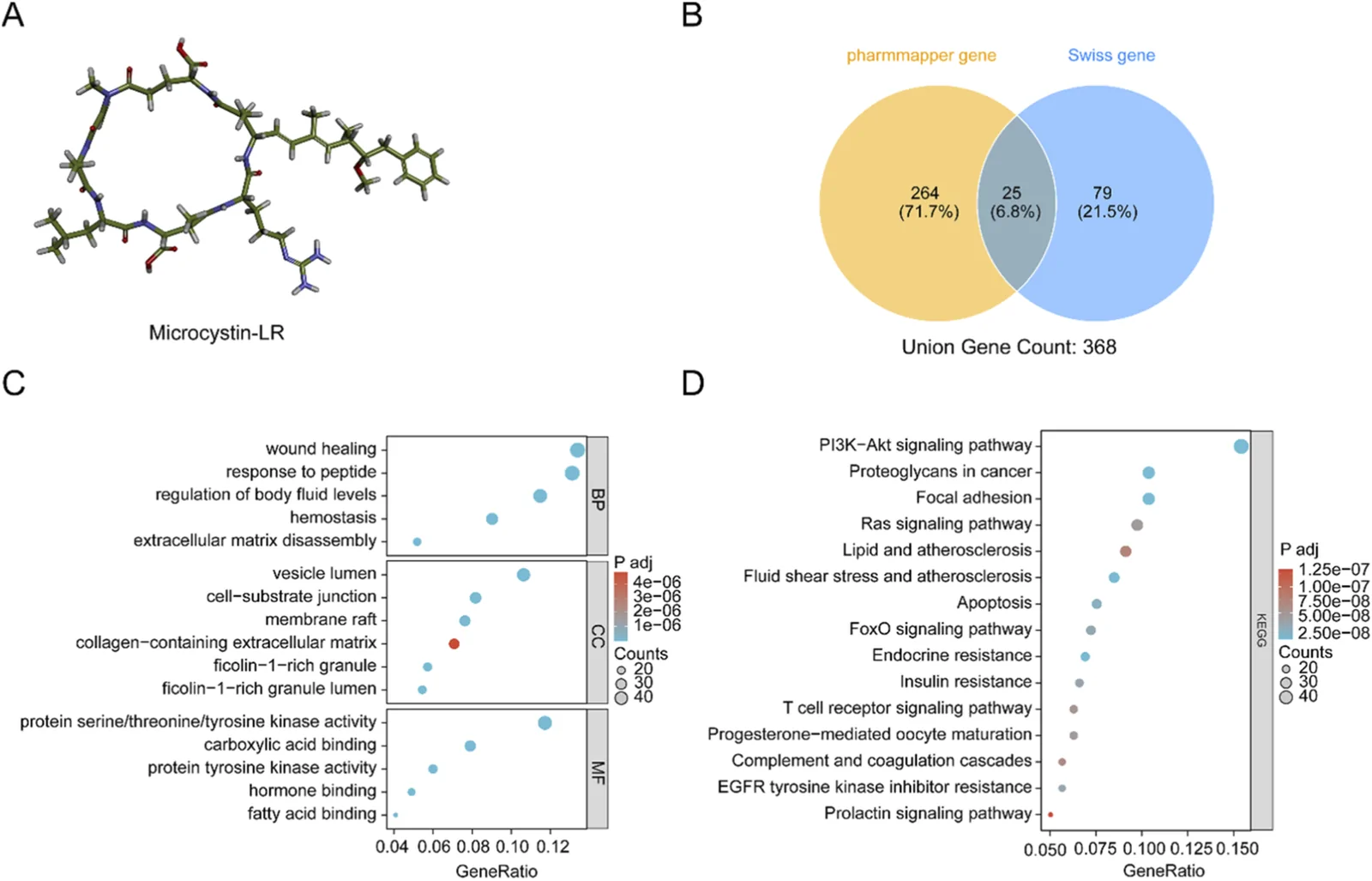
Identification and functional enrichment analysis of potential targets for MC-LR. **(A)** Three-dimensional chemical structure model of MC-LR. **(B)** Venn diagram illustrating the intersection and union of predicted MC-LR target genes retrieved from the PharmMapper and SwissTargetPrediction databases. **(C)** GO enrichment analysis of the identified union target genes displaying the top significantly enriched terms across BP, CC, and MF categories. **(D)** KEGG pathway enrichment analysis of the target genes, illustrating the top significantly enriched signaling pathways.

### 3.2 Batch correction, DEGs, and WGCNA

PCA of the five datasets showed clear batch clustering before correction (Figure 3A) that dissolved after ComBat (Figure 3B). DEG analysis identified significantly upregulated and downregulated genes. The top 25 DEGs are labeled in the volcano plot (Figure 3C).

**FIGURE 3:**
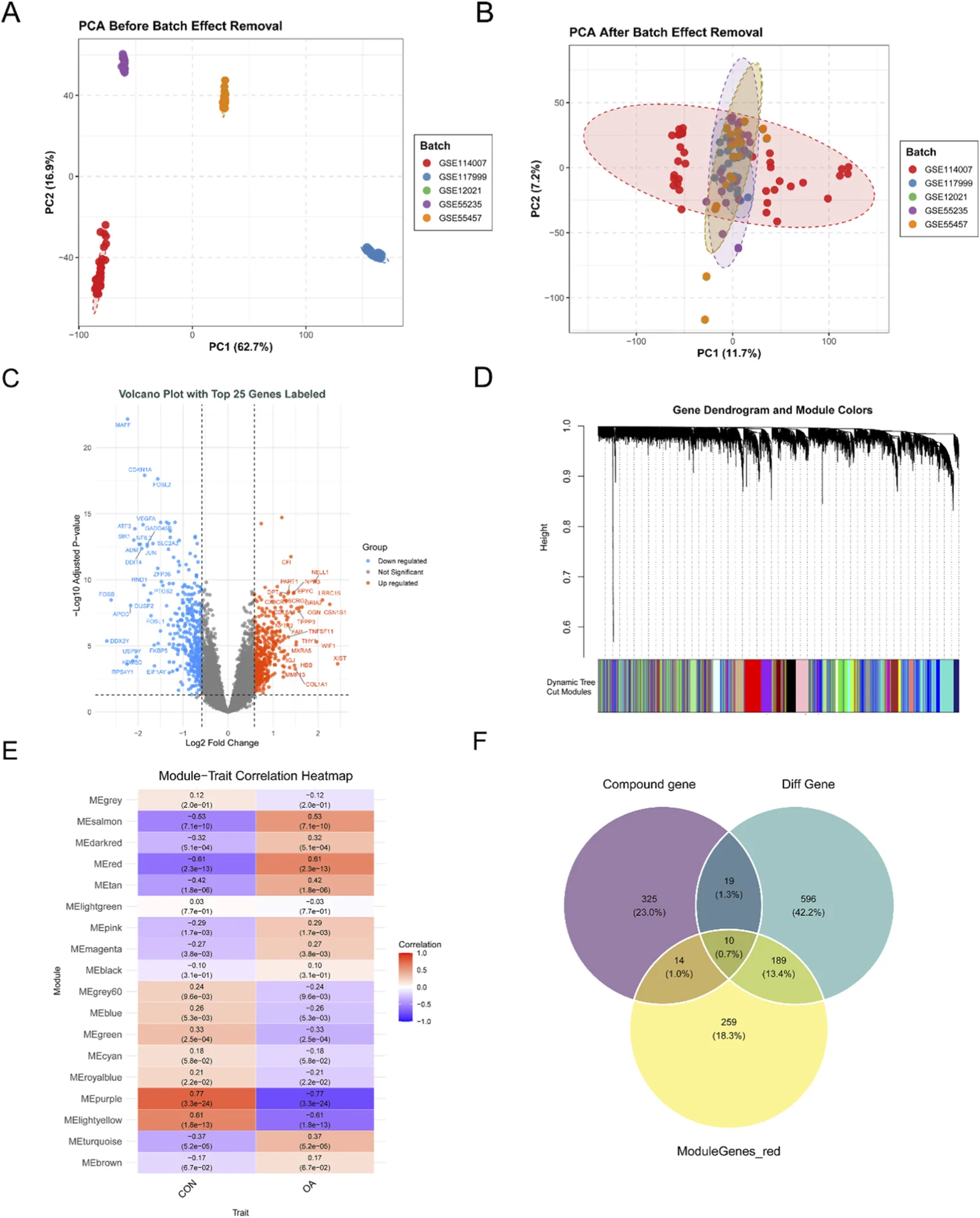
Identification of core targets using multi-dataset integration and WGCNA. **(A,B)** PCA plots of the merged datasets before and after batch effect removal. Different colors represent distinct GEO datasets. **(C)** Volcano plot of DEGs between the CON and OA groups. Red dots indicate upregulated genes, blue dots indicate downregulated genes; the top 25 significantly altered genes are labeled. **(D)** Gene dendrogram and module colors generated by WGCNA. **(E)** Module–trait correlation heatmap. The red module (MEred) shows a significant positive correlation with the disease trait. **(F)** Venn diagram illustrating the intersection of DEGs, compound targets, and module genes. A total of 10 common genes were identified.

WGCNA was used to cluster genes into color-coded modules (Figure 3D). The red module (MEred) showed the strongest positive correlation with disease (r = 0.61, p = 2.3e−13) (Figure 3E). Intersection of 368 MC-LR targets, 814 DEGs, and 472 MEred genes yielded 10 shared core candidates (Figure 3F).

### 3.3 LASSO screening and diagnostic model

LASSO with 10-fold cross-validation (Figures 4A,B) refined the 10 candidates to the following six core genes: *FAP*, *IGF1R*, *IGF1*, *MMP9*, *CRABP2*, and *ITGA4*. All six genes were significantly dysregulated between the CON and OA groups (p < 0.001; Figure 4C): *CRABP2*, *FAP*, *IGF1*, *ITGA4*, and *MMP9* were upregulated, whereas *IGF1R* was downregulated.

**FIGURE 4:**
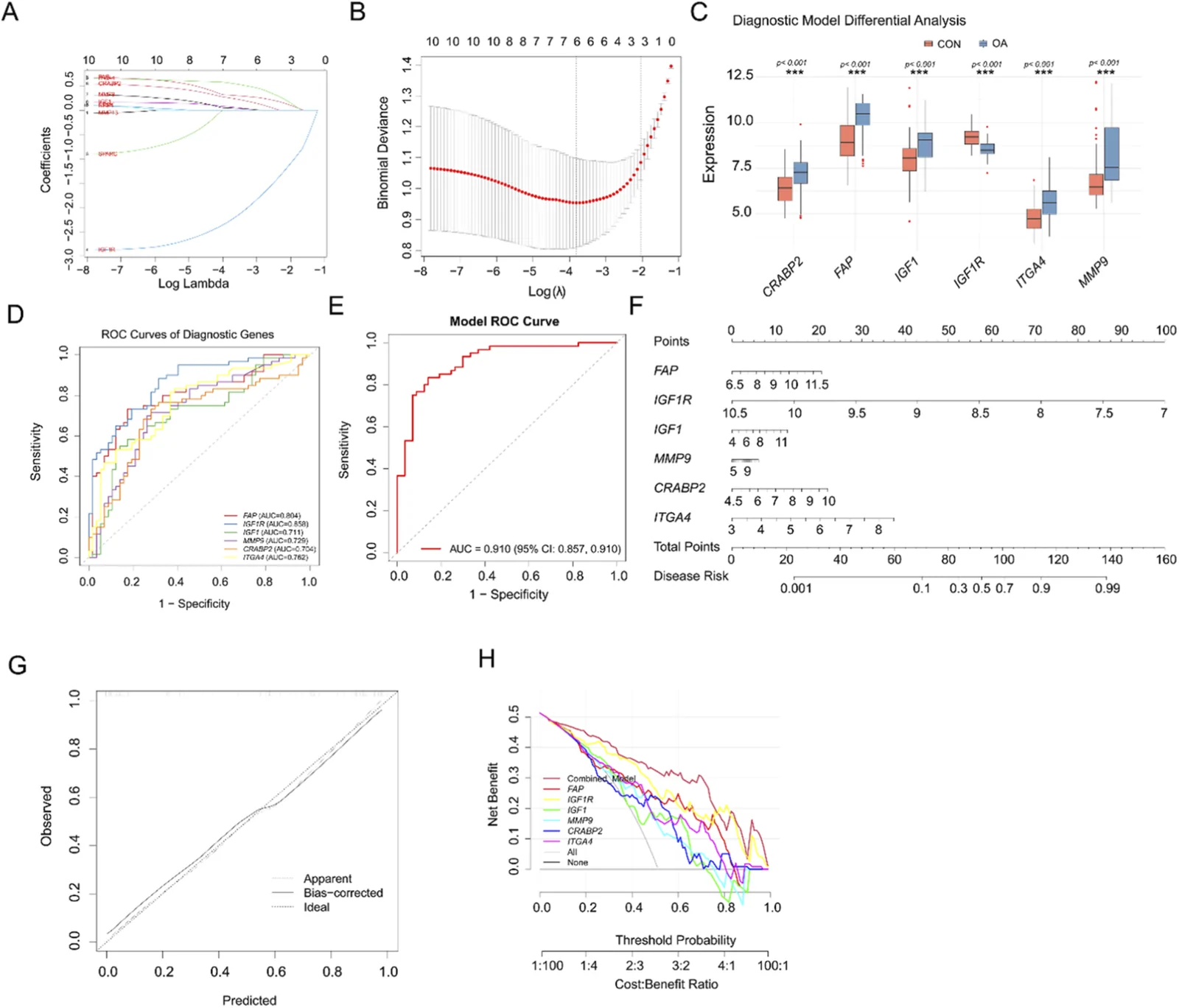
Construction and evaluation of the diagnostic model based on core biomarkers. **(A)** LASSO coefficient profiles. **(B)** Selection of the optimal λ using 10-fold cross-validation. **(C)** Differential expression analysis of the six diagnostic genes between the CON and OA groups. ***p < 0.001. **(D)** ROC curves of the individual diagnostic genes. **(E)** ROC curve of the combined diagnostic model. **(F)** Nomogram for predicting the risk for OA. **(G)** Calibration curve. **(H)** Decision curve analysis.

Individual AUCs were as follows: *IGF1R* = 0.858, *FAP* = 0.804, *ITGA4* = 0.762, *MMP9* = 0.729, *IGF1* = 0.711, and *CRABP2* = 0.704 (Figure 4D). The combined model reached AUC = 0.910 (95% confidence interval: 0.857–0.910) (Figure 4E). A nomogram was built for clinical use (Figure 4F), and the calibration curve (Figure 4G) and DCA (Figure 4H) confirmed good agreement with the observed outcomes and higher net benefit than any single biomarker.

Because the source datasets contain no MC-LR exposure information, this six-gene signature reflects general OA-associated transcriptional changes rather than an MC-LR-specific signature; its link to MC-LR is limited to the computational overlap between OA-associated genes and predicted MC-LR targets.

### 3.4 SHAP interpretation

SHAP feature importance (Figure 5A) ranked *IGF1R* first (mean |SHAP| = 0.126), followed by *CRABP2* (0.102), *FAP* (0.082), *IGF1* (0.056), *ITGA4* (0.054), and *MMP9* (0.037). The beeswarm plot (Figure 5B) showed that low IGF1R and high *CRABP2/FAP/IGF1/ITGA4/MMP9* each drove predictions toward OA, which was consistent with the differential expression pattern. Density plots (Figures 5C–H) showed clear separation of the CON and OA samples for all six genes. Dependence plots (Figures 5I–N) revealed nonlinear gene–SHAP relationships: *IGF1R* was negatively correlated (Figure 5I), whereas *CRABP2* and *FAP* were positively correlated (Figures 5J,K). Waterfall (Figure 5O) and force plots (Figure 5P) were used to visualize individual-level prediction shifts from the base to the final values, offering a personalized risk tool.

**FIGURE 5:**
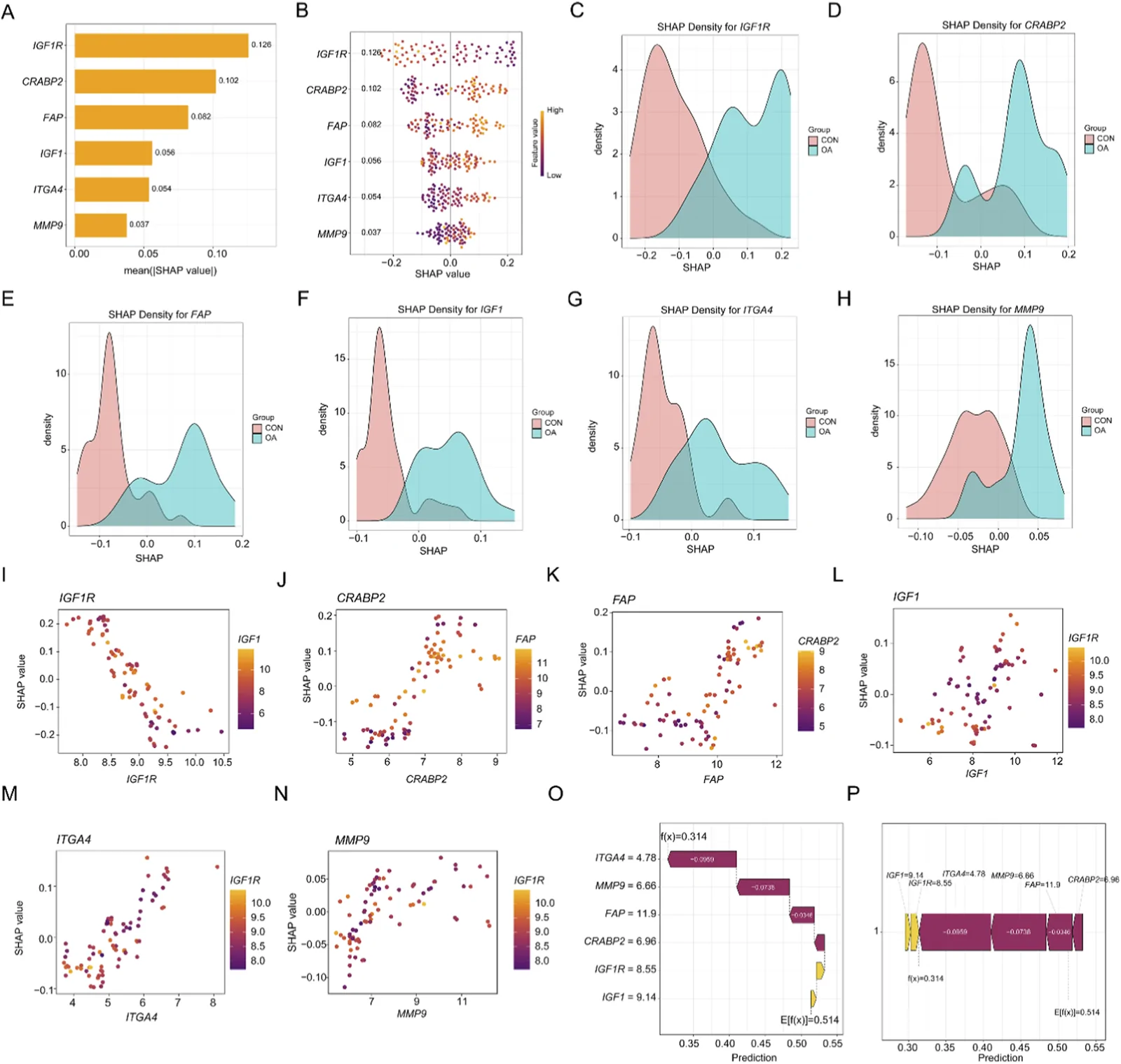
Interpretation of the diagnostic model using SHAP analysis. **(A)** SHAP feature importance bar plot. **(B)** SHAP summary plot (beeswarm plot). **(C–H)** SHAP density plots for individual genes (**(C)** *IGF1R*; **(D)** *CRABP2*; **(E)** *FAP*; **(F)** *IGF1*; **(G)** *ITGA4*; **(H)** *MMP9*). **(I–N)** SHAP dependence plots (**(I)** *IGF1R*; **(J)** *CRABP2*; **(K)** *FAP*; **(L)** *IGF1*; **(M)** *ITGA4*; **(N)** *MMP9*). **(O)** SHAP waterfall plot for a representative sample. **(P)** SHAP force plot.

### 3.5 Immune cell infiltration and gene–immune correlations

The stacked bar chart (Figure 6A) shows the overall immune cell landscape. Compared with CON tissues, OA tissues had significantly higher T cells CD8, NK cells activated, macrophage M1, macrophage M2, and mast cells activated, and lower T cells follicular helper, macrophage M0, and mast cells resting (p < 0.05; Figure 6B). The 22-cell correlation matrix (Figure 6C) reveals complex synergistic and antagonistic relationships. The correlation network (Figure 6D) and heatmap (Figure 6E) link the six biomarkers to immune cells. *MMP9* and *FAP* correlated positively with macrophage M0 (*MMP9* r = 0.66; FAP r = 0.47, p < 0.001) and negatively with plasma cells and monocytes (*MMP9* r = −0.34, p < 0.001; *FAP* r = −0.22, p < 0.05). *IGF1* was positively correlated with macrophage M2 (r = 0.21, p < 0.05). These associations indicate that the biomarkers are correlated with the immune-cell composition of the osteoarthritic joint.

**FIGURE 6:**
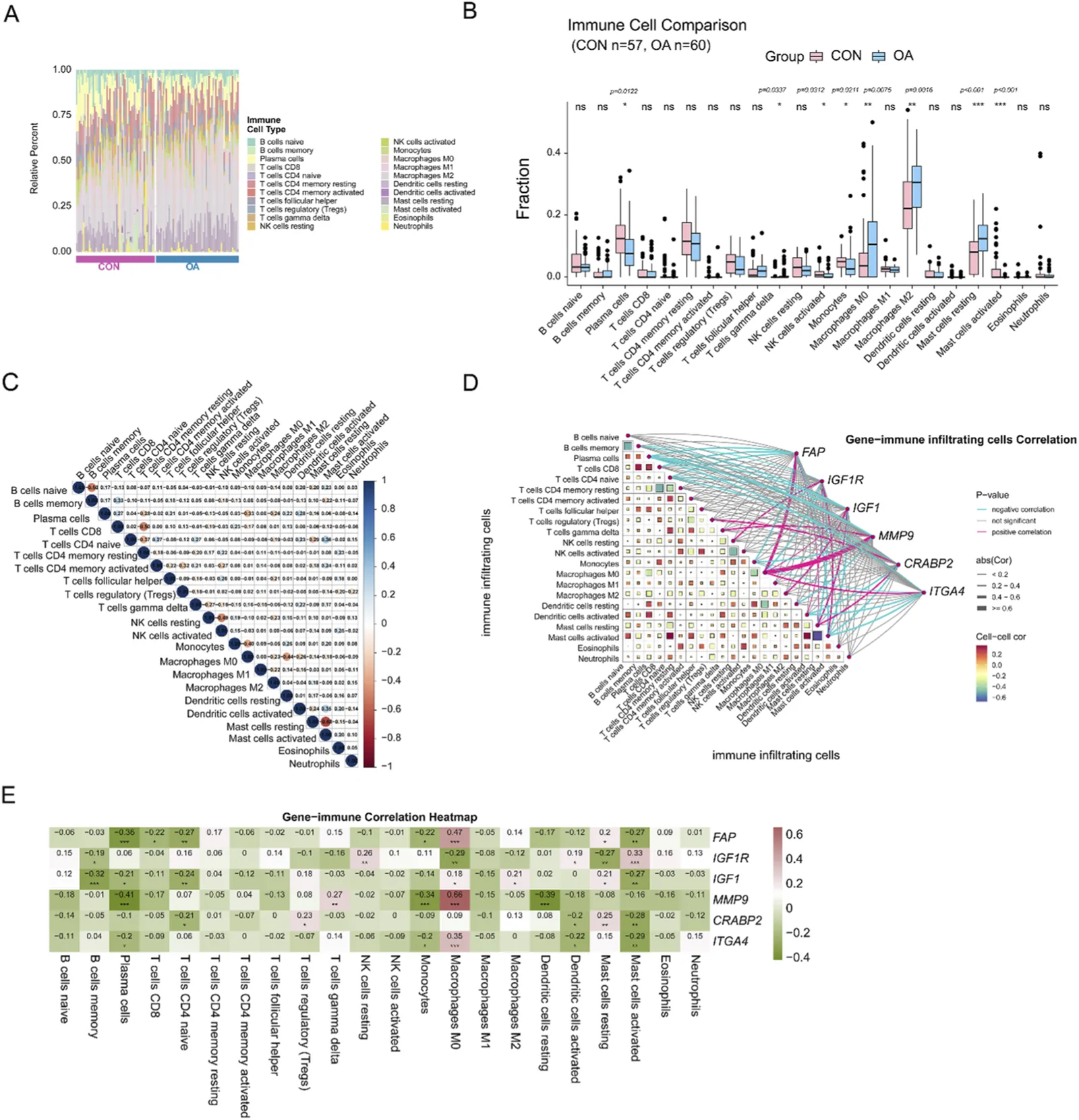
Evaluation of immune cell infiltration and its correlation with core biomarkers. **(A)** Stacked bar chart of 22 infiltrating immune cell types. **(B)** Box plots comparing differential infiltration between the CON and OA groups. ns, not significant; *p < 0.05, **p < 0.01, ***p < 0.001. **(C)** Correlation matrix of the 22 immune cell types. **(D)** Network diagram of correlations between the core genes and immune cells. **(E)** Heatmap of correlation coefficients between the six core biomarkers and 22 infiltrating immune cell types; asterisks denote statistical significance (*p < 0.05, **p < 0.01, ***p < 0.001).

### 3.6 Molecular docking and MDS with MC-LR

Docking of MC-LR with the five upregulated targets shows stable binding in all five pockets (Figures 7A–E). Key contacts include VAL-352, PHE-351, ASN-399, PHE-401, and ARG-422 in *FAP*; GLU-57 and CYS-95 in *IGF1*; GLU-63, GLU-74, and GLN-75 in *CRABP2*; TYR-44 in ITGA4; and LEU-371 and CYS-388 in *MMP9*. The lowest binding energies (Figure 7F) were *FAP* −10.3, *MMP9* −8.6, *IGF1* −7.3, *CRABP2* −6.5, and *ITGA4* −6.4 kcal/mol, with binding for *FAP* being the strongest.

**FIGURE 7:**
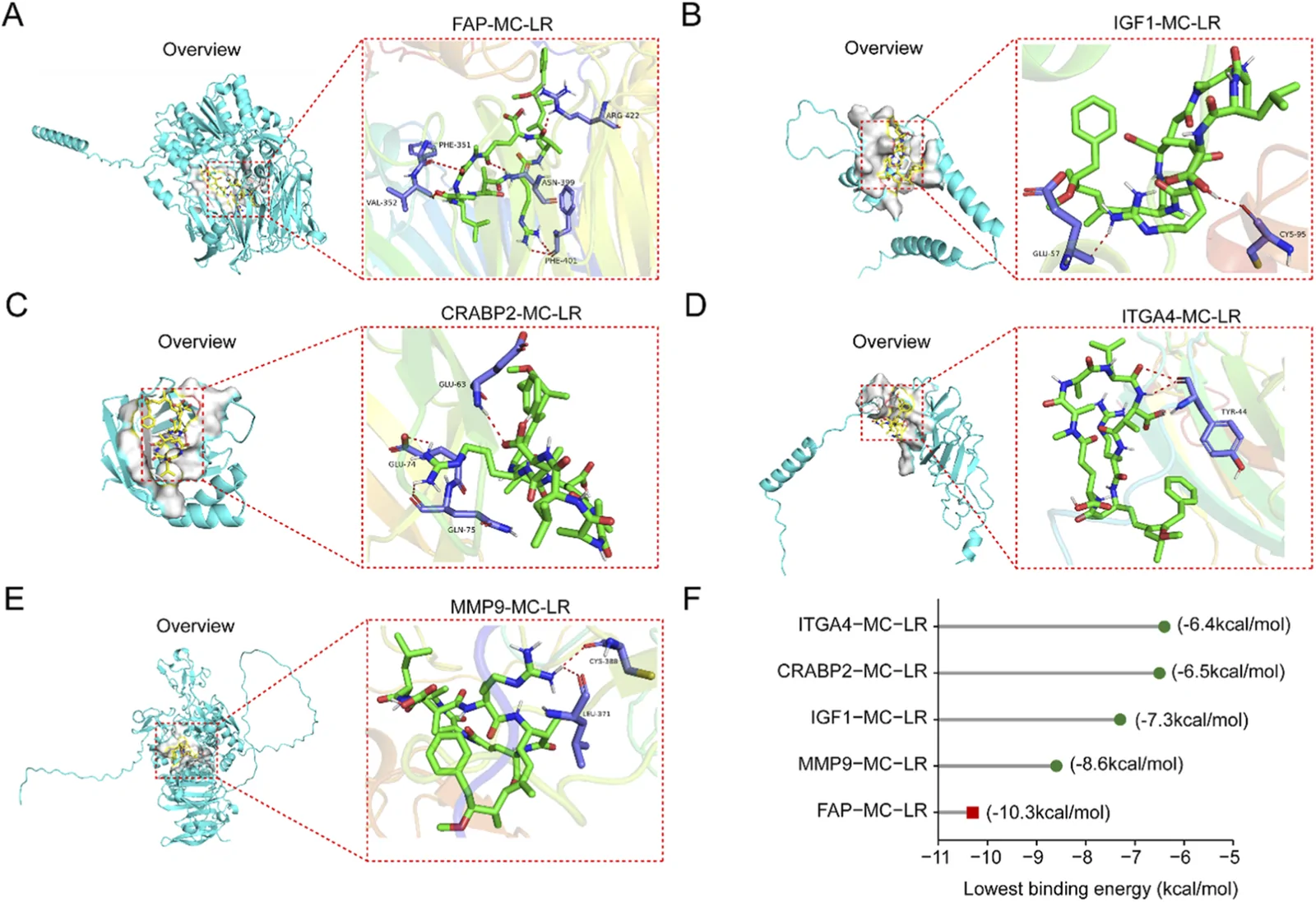
Molecular docking analysis of core targets with MC-LR. **(A–E)** Representative three-dimensional docking conformations of MC-LR with FAP **(A)**, IGF1 **(B)**, CRABP2 **(C)**, ITGA4 **(D)**, and MMP9 **(E)**. Left panels show overall structures, and right panels show magnified binding pockets. Green sticks: MC-LR. Red dashed lines: hydrogen bonds. **(F)** Lollipop chart of the lowest binding energies (kcal/mol).

Over 100-ns MDS, RMSD (Figure 8A) indicated that *FAP* and *CRABP2* reached equilibrium quickly with small fluctuations (∼0.5–0.6 nm) and that *ITGA4* stabilized after ∼25 ns. Rg values (Figure 8B) showed that FAP and *CRABP2* remained compact, whereas RMSF (Figure 8C) confirmed rigid protein cores (<0.5 nm) with peaks at flexible loops. *MMP9* maintained the highest H-bond count (6–7; Figure 8D). Gibbs FEL (Figures 8E,F) showed *CRABP2* and *FAP* with a single deep basin (thermodynamically favored) and *ITGA4* to be more diffuse. Overall, *FAP* and *MMP9* emerged as the most stable interacting targets.

**FIGURE 8:**
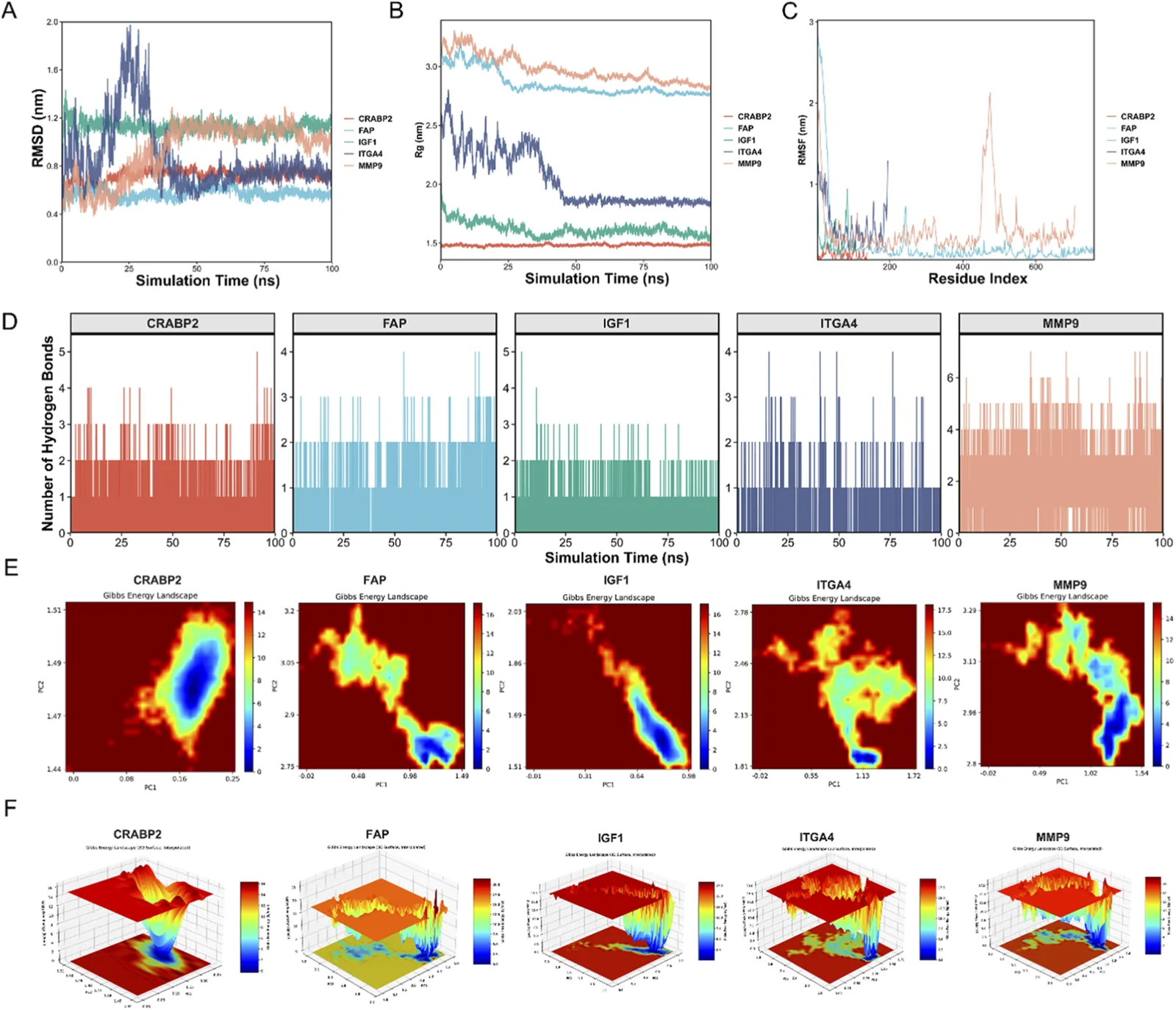
MDS analysis of the five core target–ligand complexes over 100 ns. **(A)** RMSD trajectories. **(B)** Rg trajectories. **(C)** RMSF profiles. **(D)** Intermolecular hydrogen bond counts. **(E)** Three-dimensional Gibbs free-energy landscape contour maps. **(F)** Three-dimensional Gibbs free-energy landscape surface representations.

### 3.7 Experimental validation of MC-LR-induced cytotoxicity, biomarker dysregulation, and cartilage damage

To experimentally validate our computational predictions, we evaluated the biological effects of MC-LR using both *in vitro* human chondrocytes and an *in vivo* murine model. In human chondrocytes, the CCK-8 assay revealed that MC-LR exerted a prominent dose-dependent cytotoxic effect on cell viability, yielding a calculated IC_50_ of 13.09 μM (Figure 9A). Based on this threshold, cells were treated with 13.09 μM MC-LR for 24 h to investigate downstream molecular alterations. RT-qPCR analysis demonstrated that the mRNA expressions of *CRABP2, FAP, IGF1, ITGA4,* and *MMP9* were significantly upregulated following MC-LR exposure compared with the negative control (NC) group (Figures 9B–F). These *in vitro* findings closely mirrored the differential expression patterns captured in our multi-dataset analysis, confirming that MC-LR exposure upregulates these core targets *in vitro*.

**FIGURE 9:**
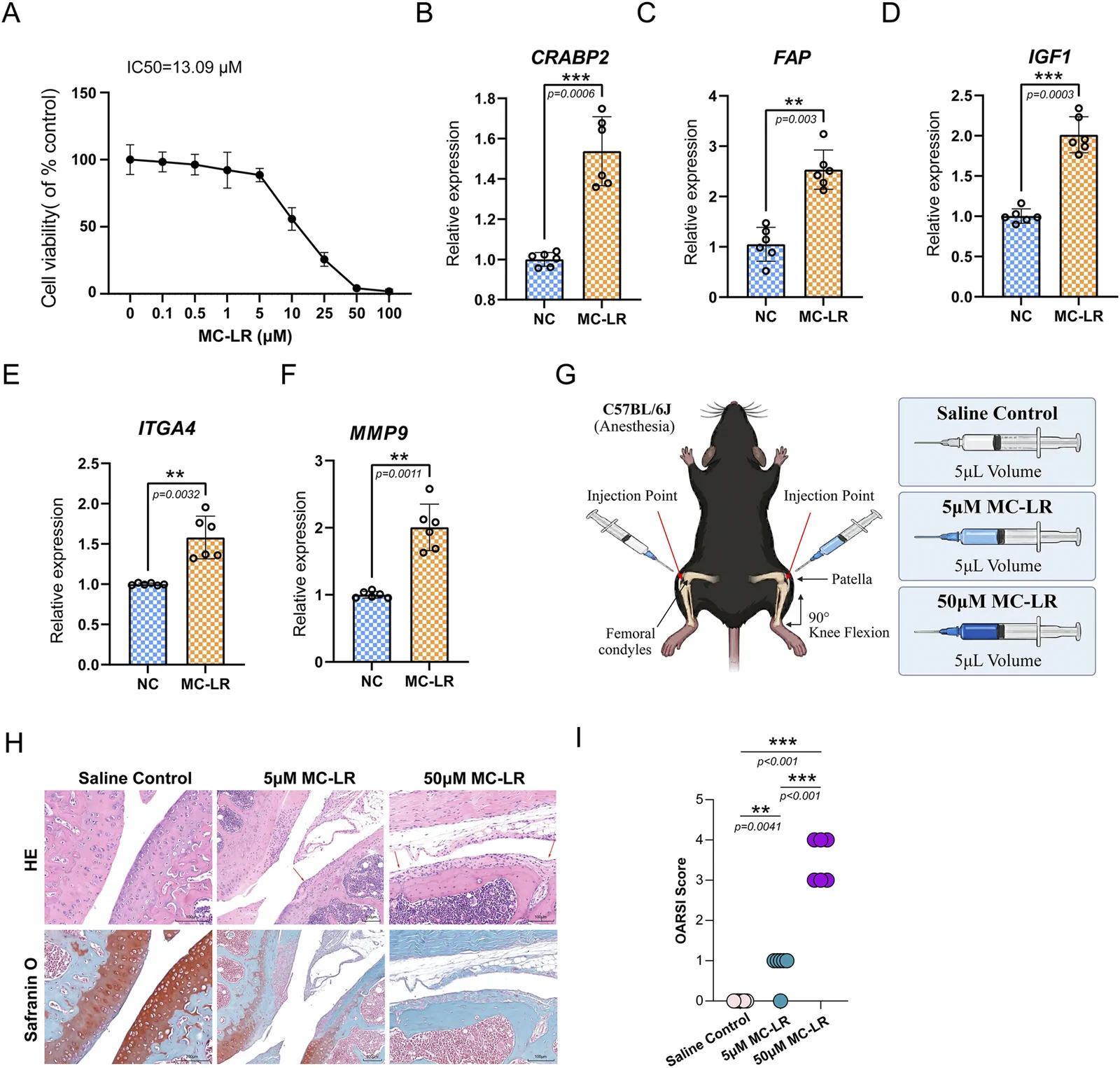
*In vitro* and *in vivo* experimental validation. **(A)** Viability of human chondrocytes treated with various concentrations of MC-LR (0–100 μM) for 24 h, assessed by CCK-8 assay (IC_50_ = 13.09 μM). **(B–F)** Relative mRNA expressions of the five core genes—*CRABP2* **(B)**, *FAP* **(C)**, *IGF1* **(D)**, *ITGA4* **(E)**, and *MMP9* **(F)**—between the negative control (NC) and MC-LR-treated human chondrocytes quantified via RT-qPCR. **(G)** Schematic of the *in vivo* experiment: male C57BL/6J mice received bilateral intra-articular knee injection (5 μL) of saline, 5 μM MC-LR, or 50 μM MC-LR (n = 3 mice, 6 joint cavities per group). **(H)** Representative hematoxylin and eosin (HE, top) and Safranin O–Fast Green (bottom) staining of knee joints from the control (NC), 5 μM, and 50 μM groups; red arrows indicate cartilage lesions. Scale bars, 100 μm. **(I)** OARSI scores of the three groups, with each point representing one knee. Data are presented as mean ± SD. **p < 0.01, ***p < 0.001.

To further assess the pathological relevance of these findings *in vivo*, male C57BL/6J mice received intra-articular knee injections of saline or MC-LR (5 or 50 μM). Histological evaluation via HE and Safranin O–Fast Green staining demonstrated that saline-treated control knees retained smooth, intact articular surfaces, regular chondrocyte arrangement, and uniform proteoglycan distribution, corresponding to an OARSI score of 0 (Figures 9H,I). In contrast, low-dose (5 μM) MC-LR treatment induced mild surface fibrillation and focal proteoglycan loss restricted to the superficial zone (OARSI score 1). High-dose (50 μM) MC-LR administration provoked severe cartilage degeneration, characterized by marked surface erosion, mid-zone fissuring, chondrocyte disorganization, and a pronounced depletion of Safranin O matrix staining (OARSI scores 3–4). Quantitatively, OARSI scores increased in a dose-dependent manner and were significantly elevated in both MC-LR exposure groups compared with the saline control (Figure 9I). Collectively, these concurrent *in vitro* and *in vivo* results indicate that MC-LR can induce chondrocyte cytotoxicity and dose-dependent cartilage matrix loss under these experimental conditions.

## 4 Discussion

MC-LR is emerging as an environmental concern because of its persistence in eutrophic freshwater and its inhibition of PP1/PP2A. Although its hepatotoxicity has been extensively characterized, its impact on the musculoskeletal system is essentially unexplored, despite the central role of PP2A in restraining pro-hypertrophic and catabolic programs in chondrocytes via the *HDAC4/MEF2/RUNX2* axis (Wei et al., 2025; Zákány et al., 2002; Kozhemyakina et al., 2009). Cartilage has limited regenerative capacity and may be especially vulnerable to chronic exposure to toxins (Skalny et al., 2024; Huey et al., 2012). To the best of our knowledge, this is the first study to systematically dissect the molecular link between MC-LR exposure and the pathogenesis of OA using an integrative framework. Using network toxicology, machine learning, MDS, and *in vitro* validation, six core biomarkers, namely, *FAP*, *IGF1R*, *IGF1*, *MMP9*, *CRABP2*, and *ITGA4*, were identified, identifying candidate OA-associated targets that are also predicted MC-LR binders, implicating PI3K-Akt, focal adhesion, and ECM remodeling as candidate pathways. These findings expand the toxicological profile of MC-LR beyond the liver and position cyanotoxin exposure, an increasingly prevalent environmental hazard driven by global warming and eutrophication (Paerl and Huisman, 2009), as a previously underrecognized contributor to degenerative joint disease.

Traditional animal- and cell-based toxicology is limited in establishing causality between pollutants and disease (Parish et al., 2020). Network toxicology complements these approaches by constructing toxin–target–disease networks (Sturla et al., 2014; Hopkins, 2008). Our 368 predicted MC-LR targets were enriched in PI3K-Akt, proteoglycans in cancer, focal adhesion, and Ras signaling pathways, all of which are central to chondrocyte biology. PI3K-Akt governs chondrocyte survival, proliferation, and matrix synthesis (Starkman et al., 2005; Sun et al., 2020), whereas focal adhesion mediates integrin-dependent mechanotransduction (Loeser, 2014). This convergence explains how chronic MC-LR exposure can predispose to degenerative joint disease, extending MC-LR toxicology beyond the liver.

Among the six genes selected by LASSO, FAP is a transmembrane serine protease involved in ECM degradation and fibrosis. It is upregulated in the synovium in OA (Bauer et al., 2006; Fan et al., 2023). *MMP9* degrades type IV collagen and gelatin and is central to cartilage destruction (Burrage et al., 2006; Zítka et al., 2010). *IGF1* promotes proteoglycan synthesis, and its overexpression in OA likely reflects an insufficient compensatory response (Loeser and Shanker, 2000; Wen et al., 2021). *CRABP2* regulates retinoic acid signaling during chondrocyte differentiation and hypertrophy (Napoli, 2017; Williams et al., 2010). *ITGA4* mediates cell–ECM adhesion and leukocyte trafficking (Miao et al., 2024). *IGF1R*, the only downregulated gene, is the receptor for IGF1-mediated chondroprotection. Its decrease may impair anabolic signaling and shift the balance toward catabolism (Wen et al., 2021; Loeser, 2009). This combination of increased ligand and reduced receptor is most consistent with functional resistance to anabolic IGF-1 signaling, in which the concurrent rise in IGF1 represents a failed compensatory response rather than restored anabolism; because this inference rests on transcript-level data, receptor-level protein and phosphorylation measurements would be needed to confirm it. This pattern aligns with the inhibition of PP2A by MC-LR, which can hyperactivate kinase signaling while disrupting receptor-mediated anabolic pathways (Reynhout and Janssens, 2019).

The combined six-biomarker model achieved an AUC of 0.910, outperforming individual genes. *IGF1R* and *CRABP2* were identified as the most influential predictors using SHAP. Calibration and DCA confirmed robust discrimination and clinical net benefit. However, the model was built on GEO datasets without MC-LR exposure information; therefore, it reflects general OA diagnostics rather than MC-LR-specific detection. Future studies using MC-LR–exposed cohorts with matched controls would enable exposure-specific tools.

CIBERSORT revealed elevated M1 and M2 macrophages and reduced M0 macrophages in OA, consistent with chronic inflammation and imbalance in macrophage polarization driving cartilage destruction (Bondeson et al., 2010). *MMP9* and *FAP* correlated positively with M0 macrophages, and *IGF1* correlated positively with M2 macrophages, suggesting that the biomarkers may help shape immune cell recruitment in joints. MC-LR activates NF-κB and MAPK signaling (Zhang et al., 2025), and its impact on macrophage-mediated joint inflammation merits further study.

Findings from molecular docking revealed strong MC-LR binding to all five targets, especially FAP (−10.3 kcal/mol). MDS confirmed stable complexes based on RMSD, RMSF, Rg, H-bond counts, and FEL, suggesting that upon binding, MC-LR may continuously interfere with these proteins and produce effects that persist beyond exposure. These interactions are *in silico* predictions; experimental validation is therefore warranted (e.g., SPR, ITC). Our findings showed good binding affinities for all complexes, particularly for MC-LR–FAP (−10.3 kcal/mol). MDS was used to further confirm the stability of these complexes, with RMSD, RMSF, and Rg values indicating persistent interactions. The stable number of hydrogen bonds and single energy clusters in the FEL plots further support our conclusion. These stable interactions at the molecular level may explain the long-term biological effects of MC-LR. Once MC-LR binds to these key proteins, it may continuously interfere with their functions, with adverse effects on the cartilage potentially persisting over the long-term even after MC-LR exposure ceases. It should be acknowledged that molecular docking and MDS represent *in silico* predictions that warrant further experimental validation using techniques such as surface plasmon resonance and isothermal titration calorimetry.

Findings from our *in vitro* experiments using human chondrocytes experimentally validated our computational findings. MC-LR exhibited pronounced cytotoxicity toward chondrocytes (IC_50_ = 13.09 μM), confirming the susceptibility of cartilage cells to the cyanotoxin. Importantly, exposure to MC-LR significantly upregulated *CRABP2*, *FAP*, *IGF1*, *ITGA4*, and *MMP9* at the mRNA level, directly mirroring the expression patterns observed in the multi-dataset bioinformatics analysis. This concordance substantially strengthens the reliability of our integrative analytical framework. However, some drawbacks should be pointed out: MC-LR levels leading to significant changes during cell culture may not directly translate to *in vivo* exposure levels (Buratti et al., 2017), and the immortalized human chondrocyte line may not completely replicate the behavior of primary articular chondrocytes. It should also be emphasized that the *in vitro* and intra-articular concentrations used here are several orders of magnitude above environmentally encountered systemic levels, and were chosen to characterize intrinsic cartilage toxicity rather than to simulate environmental exposure. Consistent with these *in vitro* observations, intra-articular MC-LR injection in mice produced dose-dependent cartilage degeneration with elevated OARSI scores on HE and Safranin O–Fast Green staining, providing *in vivo* support for a contribution of MC-LR to cartilage damage under these experimental conditions.

Integrating these findings suggests a coherent mechanistic route rather than a set of isolated associations. Following cellular uptake through organic anion-transporting polypeptides, the proximal event is MC-LR-mediated inhibition of PP1 and PP2A, the toxin’s best-characterized molecular action (MacKintosh et al., 1990). Because PP2A normally restrains the pro-hypertrophic and catabolic program of chondrocytes through the HDAC4/MEF2/RUNX2 axis and suppresses catabolic effectors such as MMP-13, its inhibition is expected to derepress precisely these pathways (Wei et al., 2025; Zákány et al., 2002; Kozhemyakina et al., 2009). The consequent net rise in substrate phosphorylation would sustain PI3K-Akt signaling, the most enriched pathway among our predicted targets and a central regulator of chondrocyte survival and matrix turnover (Starkman et al., 2005; Sun et al., 2020), while favoring the inflammatory MAPK and NF-κB activation that MC-LR is known to drive in other tissues (Zhang et al., 2025). In parallel, MC-LR induces oxidative stress and mitochondrial dysfunction (Ge et al., 2024), which amplify this inflammatory tone and further destabilize chondrocyte homeostasis. These upstream events are mirrored by the specific genes identified here: an anabolic imbalance of increased IGF1 with reduced IGF1R signaling (Loeser and Shanker, 2000; Wen et al., 2021), a CRABP2-linked shift in retinoic-acid signaling that favors hypertrophy (Napoli, 2017; Williams et al., 2010), and upregulation of the matrix-degrading effectors FAP and MMP9 that we measured directly (Fan et al., 2023; Burrage et al., 2006; Zítka et al., 2010), alongside which the canonical osteoarthritic collagenase and aggrecanases, MMP13 and ADAMTS4/5, would be predicted to act, although these were not assessed in the present study. Increased ITGA4, in turn, supports integrin-mediated adhesion and leukocyte trafficking (Loeser, 2014; Miao et al., 2024), consistent with the altered macrophage polarization seen in our immune-infiltration analysis. Because MC-LR inhibits both phosphatases and our study did not include isoform-selective inhibition or knockdown, the observed effects cannot be attributed to PP2A alone; both PP1 and PP2A regulate the serine/threonine phosphorylation that governs chondrocyte survival, cytoskeletal organization, and catabolic gene expression, and dissecting their individual contributions will require isoform-selective tools in future work. Collectively, these steps trace a plausible path from phosphatase inhibition to a net catabolic tipping of the anabolic-catabolic balance and progressive cartilage matrix loss.

Our study is the first of its kind to demonstrate the detailed molecular link between MC-LR exposure and OA. Limitations include reliance on computational and *in vitro* data with only short-term *in vivo* validation (chronic-exposure and cohort studies are required to establish causality), absence of MC-LR exposure information in GEO datasets, the use of only male mice despite well-documented sex differences in OA, so that female animals will be needed in future work to test for sex-dependent susceptibility to MC-LR, the small *in vivo* sample (n = 3 mice, 6 knees per group), which we regard as a proof-of-concept demonstration of a dose-dependent cartilage response rather than a study powered for effect-size estimation, the combination of cartilage and synovial datasets so that the signature reflects joint-level rather than cartilage-specific OA, the use of an intra-articular route and supra-environmental concentrations that model local toxicity rather than environmental exposure, and the absence of protein-level and pathway-level functional validation (including of *IGF1R* and of anabolic and catabolic markers such as *COL2A1*, *ACAN*, *MMP13*, and *ADAMTS5*), which we identify as the primary next step. Future studies should explore the role of these genes in cartilage biology, study chronic MC-LR exposure using oral or drinking-water chronic low-dose models, and develop biomarker assays for exposed populations.

## 5 Conclusion

By integrating network toxicology, machine learning, molecular docking, and MDS, six core biomarkers, namely, *FAP*, *IGF1R*, *IGF1*, *MMP9*, *CRABP2*, and *ITGA4*, were identified, linking MC-LR exposure to OA via PI3K-Akt signaling, focal adhesion, and ECM remodeling pathways. The combined diagnostic model demonstrated robust predictive performance (AUC = 0.910), whereas MDS confirmed stable thermodynamic interactions between MC-LR and these target proteins. *In vitro* experiments in human chondrocytes corroborated the computational predictions, demonstrating significant MC-LR–induced cytotoxicity and the upregulation of five key biomarkers (*FAP*, *IGF1*, *MMP9*, *CRABP2*, and *ITGA4*). Moreover, intra-articular MC-LR injection in mice induced dose-dependent cartilage degeneration with elevated OARSI scores, consistent with the *in vivo* relevance of these findings. Collectively, these findings highlight environmental cyanotoxin exposure as an underrecognized risk factor for degenerative joint disease and provide an integrative framework for investigating the musculoskeletal toxicity of related environmental pollutants.

## Data Availability

The datasets presented in this study can be found in online repositories. The names of the repository/repositories and accession number(s) can be found below: NCBI Gene Expression Omnibus (https://www.ncbi.nlm.nih.gov/geo/), GSE114007, GSE117999, GSE12021, GSE55235, and GSE55457; RCSB Protein Data Bank (https://www.rcsb.org/), 1Z68, 1GZR, 4H3X, 1CBS, and 4WJK. The processed data and analysis code are available at https://github.com/Tzf12345/TZF_bioinformatics_analysis.
